# A Novel Intronic Variant in *MED12* Associated with a Predominantly Hepatobiliary Phenotype Suggestive of Hardikar Syndrome: A Case Report and Literature Review

**DOI:** 10.3390/genes17070787

**Published:** 2026-07-09

**Authors:** Nabil El Kahy, Adib Moukarzel, Nada Assaf, Riwa Chdid, Romy Moussallem, Nabiha Salem, Alain Chebly

**Affiliations:** 1 Faculty of Medicine, Saint Joseph University of Beirut, Beirut 1104 2020, Lebanon; nabil.kahy@net.usj.edu.lb; 2 Center Jacques Loiselet for Medical Genetics and Genomics (CGGM), Saint Joseph University of Beirut, Beirut P.O. Box 17-5208, Lebanon; 3 Department of Pediatrics, Hotel-Dieu de France University Hospital, Beirut 1104 2020, Lebanon; 4 Department of Pathology and Laboratory Medicine, American University of Beirut Medical Center (AUBMC), Beirut 1107 2020, Lebanon

**Keywords:** Hardikar syndrome, MED12, rare disease, cholestasis, genotype-phenotype correlation, segregation analysis, whole-exome sequencing

## Abstract

Background/Objectives: Hardikar syndrome (HDKR) is an X-linked dominant disorder caused by pathogenic variants in the Mediator complex subunit 12 (*MED12)* gene, predominantly affecting females. It is characterized by multisystem congenital anomalies involving the foregut, biliary tract, craniofacial structures, eyes, skeleton, and genitourinary system, with generally preserved neurodevelopment. Only 34 cases have been reported to date, and most exhibit multiple congenital anomalies. We describe a female infant who presented with progressive cholestatic liver disease and complex hepatobiliary malformations, including an absent gallbladder and paucity of bile ducts, with unremarkable prenatal imaging. The clinical course was notable for hepatosplenomegaly, markedly elevated total bile acids, portal hypertension with esophageal varices, and eventual liver failure. Methods: Whole-exome sequencing (WES) was performed to investigate the underlying genetic etiology, followed by parental segregation analysis using Sanger sequencing to confirm and characterize the identified variant. Results: WES identified a novel de novo intronic heterozygous variant in *MED12* (c.3868-5C>G). Unlike most previously reported cases, the predominant and early manifestation in our case was severe hepatobiliary disease with limited additional anomalies, suggesting possible phenotypic variability within the *MED12*-related Hardikar syndrome spectrum. The identified *MED12* variant is classified as a variant of uncertain significance (VUS). Conclusions: This case underscores the diagnostic utility of WES in infants with unexplained cholestasis, highlights the importance of considering noncoding variants, and illustrates the value of reporting well-characterized patients carrying novel VUS, thereby contributing to the growing body of clinical and molecular evidence on *MED12*-related Hardikar syndrome.

## 1. Introduction

Hardikar Syndrome (HDKR) (MIM 301068) is an ultra-rare female-specific genetic disorder characterized by multiple congenital anomalies. HDKR is an X-linked dominant disorder, caused by heterozygous pathogenic variants in the *MED12* gene (MIM 300188). To the best of our knowledge, only 34 cases are documented in the medical literature to date, and the true prevalence of HDKR remains unknown [1].

The *MED12* gene, located on the X chromosome (Xq13.1) [2], encodes a subunit of the Mediator complex that serves as an interface between transcription factors and RNA polymerase II, thereby regulating their respective functions and transcriptional activity [3]. The human Mediator complex is a ~1.4 MDa multiprotein assembly playing a central role in transcriptional regulation. It facilitates the interaction between DNA-binding transcription factors and RNA polymerase II [4]. Additionally, MED12 interacts with chromatin and transcription factors, thereby regulating transcriptional activity [5,6,7]. MED12 deficiency impairs ciliogenesis and reduces Hedgehog and YAP pathway activity, suggesting that disruption of these signaling mechanisms may contribute to the HDKR phenotype [8].

Germline pathogenic variants in *MED12* are associated with a spectrum of overlapping X-linked disorders, including HDKR (MIM 301068), Lujan–Fryns syndrome (MIM 309520), X-linked Ohdo syndrome (MIM 300895), Opitz–Kaveggia syndrome/FG syndrome-1 (MIM 305450), and nonspecific intellectual disability (NSID). Missense variants are predominantly identified in males with FG syndrome-1, Lujan–Fryns syndrome, X-linked Ohdo syndrome, or nonspecific intellectual disability [9]. In contrast, truncating loss-of-function variants appear to be poorly tolerated in males and are presumed to result in embryonic lethality. HDKR is mainly associated with de novo truncating variants, most commonly nonsense or frameshift [10].

The clinical spectrum of HDKR includes foregut malformations, intestinal malrotation, hepatobiliary disease, genitourinary anomalies, cleft lip and/or palate, and pigmentary retinopathy. Some affected individuals also present with congenital cardiac defects or vascular abnormalities, such as aortic coarctation and carotid or intracranial aneurysms. Neurodevelopment and cognitive function are generally preserved despite multisystem involvement [1]. Recently, the phenotypic variability of HDKR has expanded, with some affected females reported to present with congenital diaphragmatic hernia [10] or pulmonary hypoplasia [11]. This phenotypic heterogeneity in females was suggested to be attributed to skewed X-chromosome inactivation (XCI) across different tissues [1].

The diagnosis of HDKR can be particularly challenging, given the broad phenotypic and molecular spectrum of *MED12*-related disorders [8], as well as the intra-syndromic variability observed between affected individuals [11]. In this context, the use of whole-exome sequencing (WES) represents a valuable tool to help overcome these clinical and phenotypic diagnostic challenges.

We hereby report a Lebanese infant with a phenotype highly suggestive of Hardikar syndrome in whom WES identified a novel de novo intronic *MED12* variant currently classified as a VUS. This report expands the clinical and molecular spectrum of MED12-related Hardikar syndrome while contributing evidence that may assist future variant interpretation. Additionally, we provide a review of the literature summarizing all reported cases of HDKR.

## 2. Materials and Methods

### 2.1. Literature Review

A literature review was conducted using PubMed. The following search terms were used to identify eligible articles: “Hardikar syndrome”, “MED12 AND syndrome”, “Hardikar syndrome AND MED12”, and “MED12-related disorder”. The search was limited to English-language articles reporting clinical, genetic, and molecular findings related to Hardikar syndrome and the MED12-related Hardikar syndrome spectrum. Studies were screened based on their titles and abstracts, and full texts were reviewed when available and relevant. 

Germline *MED12* variants classified as pathogenic or likely pathogenic were compiled from published reports and curated variant databases, including the Human Gene Mutation Database (HGMD Professional), ClinVar, and DECIPHER. Database interrogation was last performed on 24 June 2026.

### 2.2. Patient

**Figure 1:**
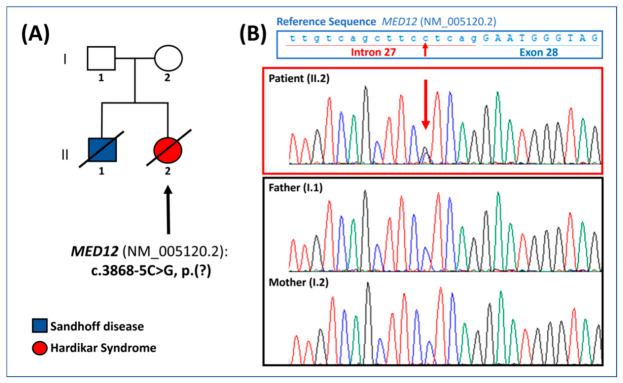
(**A**) Family pedigree. The proband (II.2, arrow) is affected with Hardikar syndrome (red). Her brother (II.1) was affected with Sandhoff disease (blue). Both parents are unaffected. (**B**) Sanger sequencing chromatograms showing the novel *MED12* variant c.3868-5C>G (NM_005120.3) in the proband. The variant is absent in both parents, confirming its de novo occurrence. The position of the variant at the intron 27–exon 28 boundary is indicated.

The proband is a 3-year-old Lebanese girl (Figure 1A, II.2), the second child from a non-consanguineous marriage. Due to her symptoms (Results section), the patient (Figure 1A, II.2) and her parents (Figure 1A, I.1 and I.2) were referred for genetic evaluation to the Centre Jacques Loiselet for Medical Genetics and Genomics (CGGM) at the Faculty of Medicine at Saint Joseph University of Beirut (USJ), Lebanon. There was no known family history of similar conditions. However, her family history was notable for the presence of Sandhoff disease (MIM #268800) due to compound heterozygous pathogenic variants in the *HEXB* gene (MIM #606873) (NM_000521.3: c.850C>T and c.1082+5G>A) previously documented in her brother, deceased at the age of 18 months (Figure 1, II.1). Her parents were molecularly confirmed to be heterozygote carriers of the identified *HEXB* variants. Genetic counseling was provided, and informed consent for genetic analyses and publication was obtained from both parents.

### 2.3. Whole Exome Sequencing (WES)

WES was performed, guided by the following Human Phenotype Ontology (HPO) terms: Cholestasis (HP:0001396), cirrhosis (HP:0001394) and hepatic failure (HP:0001399). Genomic DNA was enzymatically fragmented, and target regions were enriched using DNA capture probes covering approximately 41 Mb of the human coding exome, targeting >98% of coding RefSeq regions (GRCh37/hg19), as well as the mitochondrial genome. Sequencing was performed on an Illumina platform, achieving a mean coverage depth sufficient to ensure ≥20× coverage for 99.52% of targeted nucleotides. An in-house bioinformatics pipeline was applied, including read alignment to GRCh37/hg19 genome assembly and the revised Cambridge Reference Sequence (rCRS) of the Human Mitochondrial DNA (NC_012920), followed by variant calling, annotation, and comprehensive variant filtering.

### 2.4. Sanger Sequencing

A subsequent familial segregation study was performed using standard PCR followed by Sanger sequencing. The following set of primers were used to amplify the region of interest in *MED12* (NM_005120.3): Forward primer: 5′-TGATCACACCAGCTCCCTAC-3′ and Reverse primer: 5′-ACTGCCCCTCTCCTTTTCTC-3′. PCR products were purified and used for bidirectional Sanger sequencing on an ABI 3500 sequencer (Applied Biosystems, Foster City, CA, USA).

## 3. Results

### 3.1. Literature Review

From our literature search, 12 published reports comprising 33 individuals with clinical and/or molecular findings consistent with the *MED12*-related Hardikar syndrome spectrum were considered eligible. The reported molecular spectrum was predominantly composed of heterozygous *MED12* loss-of-function variants, including nonsense, frameshift, splice-region, and copy-number variants. Most variants occurred de novo, although mosaicism and maternal transmission have also been reported.

In addition, one female individual registered in the DECIPHER database (ID 482687) carried a de novo heterozygous intragenic *MED12* deletion (GRCh38: chrX: (71129456_71131855)x1). Her phenotype included intrahepatic biliary atresia, impaired liver function, cardiac and pulmonary abnormalities, urogenital anomalies, mild microcephaly, abducens palsy, and global developmental delay.

The two original cases described by Hardikar et al. were excluded because no molecular diagnosis was available. Including the present patient, a total of 35 individuals were analyzed and assigned case numbers 1–35. The clinical and molecular findings are summarized in Table 1 and Table 2.

### 3.2. Clinical Presentation

Prenatal ultrasounds were unremarkable, with no reported intrauterine anomalies in the proband. At birth, laboratory evaluation revealed elevated liver enzymes: AST 331U/L, ALT 96U/L, GGT 24U/L, and alkaline phosphatase 237U/L. On day 4 of life, conjugated hyperbilirubinemia was documented (12.92 mg/dL). Congenital coccygeal agenesis was identified at 22 months of age.

Abdominal ultrasound performed at 22 days of life demonstrated hepatomegaly with a liver measuring 7.2 cm in maximal height, an absence of the gallbladder, and non-visualization of the intrahepatic and extrahepatic bile ducts, including the common bile duct. No triangular cord sign was identified. The spleen was normal in size (5.5 cm).

After excluding biliary atresia, based on the overall clinical, imaging, and histopathological findings, microscopic examination of liver biopsy performed at 2 months of age revealed cholestasis with paucity of intralobular bile ducts, portal fibrosis with a few septa in the absence of cirrhosis, mild intrahepatocytic and Kupffer cell hemosiderosis, and absence of interlobular bile ducts in more than half of the portal tracts. Persistent worsening of hepatomegaly was noted on follow-up by serial imaging, reaching 12 cm at 19 months of age, and 15.2 cm at 3 years and 2 months.

At 3 years of age, the patient exhibited short stature (height: 88 cm, <3rd percentile) with relatively preserved weight (16 kg, 75th percentile). Clinical examination revealed symptoms of malnutrition and advanced liver disease, including severe jaundice, abdominal collateral venous circulation, marked ascites, increased abdominal circumference (68 cm), and mild edema of the lower extremities. The patient’s relatively preserved weight was likely influenced by ascites related to advanced liver disease. Signs of portal hypertension were suggested by the detection of esophageal varices on upper gastrointestinal endoscopy. The parents reported recurrent upper respiratory tract infections associated with wheezing. No formal immunological evaluation was performed; however, WES did not identify any pathogenic or likely pathogenic variants in genes associated with primary immunodeficiency. The list of analyzed genes was based on the recommendations of the International Union of Immunological Societies (IUIS) for the genetic diagnosis of inborn errors of immunity.

Progressive clinical deterioration, including the development of splenomegaly (16.2 cm), ultimately led to consideration of liver transplantation. However, the patient developed unresolved septicemia caused by *Pasteurella multocida* and died at the age of 3 years and 8 months before liver transplantation could be performed and before the genetic test results became available.

### 3.3. Molecular Results

Given the family history of autosomal recessive Sandhoff disease, targeted sequencing of the previously identified *HEXB* pathogenic variants was initially performed. Carriership of the familial variants was not detected in the proband with subsequent exclusion of Sandhoff disease as an explanation for the clinical findings. Consequently, broader genetic testing was considered, and WES was performed.

A novel heterozygous intronic variant was identified in the *MED12* gene (NM_005120.3:c.3868-5C>G) and subsequently confirmed by Sanger sequencing. This variant affects a nucleotide upstream of the canonical splice-site and is predicted to affect exon splicing. It is absent from population databases, including gnomAD, the Exome Sequencing Project (ESP), and the 1000 Genomes Project. To the best of our knowledge, this variant has not been previously reported in the literature. In silico prediction tools support a deleterious effect on protein function. SpliceAI indicates a significant probability of splice acceptor loss (Δ score = 0.60), suggesting disruption of the native acceptor site and potential alteration of normal *MED12* pre-mRNA splicing. The database splicing consensus single nucleotide variant (dbscSNV) classifies this variant as Pathogenic Moderate, with a score of 0.9995 (version v1.1). According to the ACMG/AMP/ClinGen SVI guidelines, the c.3868-5C>G variant is currently classified as a variant of uncertain significance (VUS), with moderate evidence supporting pathogenicity (PP3, PM2). No additional clinically relevant sequence variants, copy number variants, or structural variants related to the patient’s phenotype were identified by WES.

Segregation analysis by Sanger sequencing indicated the absence of this variant in both parents (Figure 1B), consistent with a de novo occurrence (PS2). In view of the rapid disease progression and death, subsequent RNA analysis could not be performed, preventing assessment of the variant’s effect on the transcript.

Altogether, the clinical and molecular findings are compatible with a MED12-related disorder within the Hardikar syndrome spectrum.

## 4. Discussion

HDKR is an ultra-rare X-linked dominant multisystem disorder caused by germline pathogenic variants in the *MED12* gene [1]. To date, the majority of reported HDKR cases result from de novo nonsense or frameshift variants leading to loss of function [10,12]. In contrast, intronic variants remain less frequently observed in the literature, and their contribution to the molecular landscape of HDKR is not yet well defined. The identification of a novel de novo intronic *MED12* variant in our patient expands the molecular and clinical spectrum of this syndrome.

**Figure 2:**
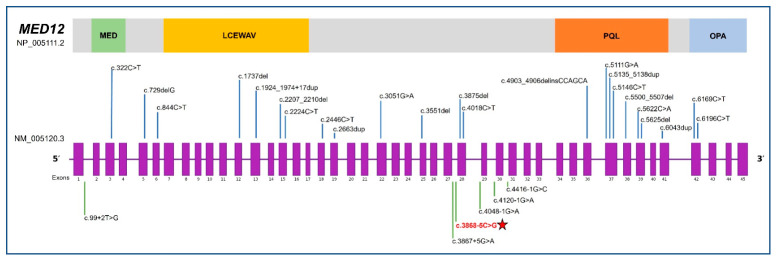
Integrated schematic representation of the *MED12* gene (NM_005120.3) and its encoded protein (NP_005111.2), showing the location of previously reported *MED12* variants associated with Hardikar syndrome. The 45 exons (purple boxes) and intervening introns (purple lines) are represented schematically. Exonic variants, including substitutions and small insertions/deletions, are indicated above the gene structure (blue lines), whereas intronic splice-region variants are shown below (green lines). The novel splice-region variant identified in the present study, c.3868-5C>G, is highlighted with a red star. The principal functional domains of the MED12 protein are shown above the gene structure: MED (Mediator complex subunit 12 domain), LCEWAV (Leu-Cys-Glu-Trp-Ala-Val conserved motif), PQL (proline-, glutamine-, and leucine-rich domain), and OPA (opa/polyglutamine-rich domain).

*MED12* encodes a critical component of the Mediator kinase module, which regulates transcriptional activity through interaction with RNA polymerase II and multiple signaling pathways [3]. Disruption of *MED12* function has been linked to impaired ciliogenesis and altered Hedgehog and YAP signaling, mechanisms that plausibly contribute to the complex developmental phenotype observed in HDKR [8]. While most previously reported variants directly truncate the protein (Figure 2); intronic variants, such as the one identified in our patient, may exert pathogenic effects through aberrant RNA splicing, potentially resulting in exon skipping, intron retention, or cryptic splice site activation. Variants located at the −5 position relative to the splice acceptor site may have variable effects on splicing efficiency, potentially allowing partial preservation of normal transcripts. This residual splicing activity could contribute to phenotypic variability and may partly explain the relatively restricted clinical presentation observed in our patient. Additionally, the presence of multiple MED12 transcript isoforms may modulate the functional impact of such splice variants, further contributing to variability in clinical expression.

To date, few studies have reported data on the molecular and clinical aspects of HDKR. Only 34 previous cases have been documented, highlighting the rarity of the syndrome and the need for more research on HDKR. Table 2 summarizes the molecular results of *MED12* variants in all HDKR cases reported in the literature including our patient (Table 2).

Functional analyses would have been valuable to assess the biological impact of the identified variant. However, RNA or protein studies could not be performed because the patient had passed away, representing an important limitation of the present study. Functional validation could, in principle, be pursued using minigene splicing assays or RNA-based approaches to assess the impact of the variant on *MED12* splicing. Although the variant occurred de novo (PS2), was absent from population databases (PM2), and showed computational evidence supporting a potential effect on splicing (PP3), computational predictions alone cannot establish pathogenicity, particularly for non-canonical splice-region variants. Consequently, the variant is conservatively classified as a VUS. Nevertheless, documenting such rare non-canonical splice variants remains important to facilitate future genotype–phenotype correlations and potential reinterpretation as additional functional or clinical evidence becomes available.

Although the identified *MED12* variant currently remains classified as a VUS according to ACMG criteria, reporting carefully phenotyped patients carrying novel VUS is an important component of the iterative process of clinical variant interpretation. Such reports contribute to evidence accumulation by documenting genotype–phenotype associations and may ultimately support variant reclassification when additional unrelated patients or functional studies become available. Therefore, we consider the present case valuable as a contribution to the expanding molecular and phenotypic spectrum of *MED12*-related Hardikar syndrome while acknowledging that definitive pathogenicity cannot yet be established.

In contrast to most published cases of HDKR, which typically display a broad constellation of multisystem anomalies including orofacial clefting, pigmentary retinopathy, cardiovascular and genitourinary malformations along with biliary anomalies [8], our patient’s phenotype was most notable for severe hepatobiliary disease including biliary tract malformation, cholestasis, and absent gallbladder. Additional findings included coccygeal agenesis, possible immunodeficiency, suggested by recurrent respiratory infections and sepsis. Importantly, regular prenatal screening was unremarkable, emphasizing the challenge of early detection in atypical presentations. The described absence of classical extra-hepatic features in our case and the predominantly hepatobiliary involvement with limited extra-hepatic features suggest a milder or variable expressivity of HDKR, which may be attributed to tissue-specific skewed XCI or differential nonsense-mediated decay of MED12 transcripts, phenomena increasingly recognized to contribute to the phenotypic heterogeneity of *MED12*-related disorders [1,22]. Finally, the early mortality in our patient, driven by progression of liver disease and inability to undergo timely transplant, underscores the severity of hepatobiliary progression in isolated presentations and reinforces the importance of genetic diagnosis to inform prognosis and management decisions.

Interestingly, some MED12 variants, such as c.6169C>T, c.5111G>A and c.844C>T (Table 2), have been reported in multiple independent cases across different studies, suggesting possible recurrent variation within the *MED12*-related Hardikar syndrome spectrum.

Although a recent review published in March 2026 provided a comprehensive overview of the clinical and molecular spectrum of Hardikar syndrome [17], two subsequently identified MED12-related cases with features compatible with the Hardikar spectrum were not included [11,12]. Together with the present case, these additional reports warranted an updated literature review, providing a more comprehensive and up-to-date synthesis of the clinical manifestations and molecular findings associated with this rare disorder.

In cases of early-onset cholestasis with hepatobiliary anomalies, several differential diagnoses should be considered. These include biliary atresia, Alagille syndrome, progressive familial intrahepatic cholestasis (PFIC), and mitochondrial hepatopathies. The absence of characteristic features of these conditions, combined with the genetic findings, was suggestive of a *MED12*-related disorder in our patient. This highlights the importance of comprehensive genomic evaluation in complex and atypical presentations.

The expanding application of molecular diagnostics increasingly reveals rare noncoding variants that would previously have remained undetected. Our findings underscore the importance of carefully evaluating intronic regions while sequencing the *MED12* gene, particularly in females presenting with features suggestive of typical or atypical HDKR. Continued analysis of such variants is essential to refine genotype–phenotype correlations and to improve variant classification frameworks for *MED12*-related disorders.

In conclusion, this study highlights the value of WES in diagnosing rare disorders with overlapping clinical features. Although the identified de novo intronic *MED12* variant remains classified as a VUS, the present report expands the clinical and molecular spectrum of *MED12*-related Hardikar syndrome and emphasizes the importance of documenting well-characterized rare cases to facilitate future evidence accumulation and variant interpretation. Additional unrelated patients and/or functional studies will be required to confirm pathogenicity and potentially support future variant reclassification.

## Figures and Tables

**Table 1:** Clinical overview of all reported cases of Hardikar syndrome (HDKR).

CASES 1-35									
Clinical Feature/Field	CASE 1	CASE 2	CASE 3	CASE 4	CASE 5	CASE 6	CASE 7	CASE 8	CASE 9
**Sex**	F	F	F	F	F	F	F	F	F
**Age at diagnosis**	7 years	43 years	8 years	4 months	5 months	11 years	1 year	7 years	5 years
**Age at last evaluation**	7 years	43 years	8 years	4 months	5 months	11 years	1 year	7 years	5 years
**Development—intellectual (HP:0001249)**	Normal	Normal	Normal	Normal	NA	Normal	NA	Normal	Normal
**Development—motor (HP:0001263)**	Normal	Normal	Normal	Normal	NA	Normal	NA	Normal	Normal
**Development—language (HP:0000750)**	Normal	Normal	Normal	Normal	NA	Normal	NA	Normal	Normal
**Cleft lip (HP:0410030)**	No	Yes, not specified	No	Yes, unilateral, not specified	No	No	No	No	Yes, not specified
**Cleft palate (HP:0000175)**	No	Yes, not specified	No	Yes, unilateral, not specified	No	Yes, not specified	No	No	Yes, not specified
**Hearing impairment (HP:0000365)**	NA	Yes, unspecified	NA	NA	NA	NA	NA	NA	Yes, right sided, severe, type not specified
**Pigmentary retinopathy (HP:0000580)**	No	NA	Yes	No	NA	Yes	Yes	Yes	Yes
**GI—Intestinal malrotation (HP:0002566)**	Yes	No	No	No	No	Yes	No	Yes	Yes
**GI—cholangitis (HP:0030151)**	No	NA	NA	No	NA	NA	NA	NA	NA
**GI—cholestasis (HP:0001396)**	No	NA	NA	No	NA	NA	NA	NA	NA
**GI—choledochal cyst (HP:0100890)**	No	NA	NA	No	NA	NA	Yes	NA	Yes
**GI—hepatomegaly (HP:0002240)**	NA	NA	NA	NA	NA	NA	NA	NA	NA
**GI—splenomegaly (HP:0001744)**	NA	NA	NA	NA	NA	NA	NA	NA	NA
**GI—absent gallbladder (HP:0011467)**	No	No	No	No	No	No	No	No	No
**GI—liver fibrosis (HP:0001395)**	NA	NA	NA	NA	NA	NA	NA	NA	NA
**GI—jaundice (HP:0000952)**	No	NA	NA	No	NA	NA	NA	NA	NA
**GI—Biliary atresia (HP:0005912)**	No	NA	NA	No	NA	Yes	NA	NA (biliary dysgenesis)	Yes
**GI—obstructive liver disease (HP:0005230)**	No	NA	NA	No	NA	NA	NA	NA	NA
**GI—liver biopsy/histology notes**	NA	NA	NA	NA	Dilated infrahepatic ducts	NA	NA	NA	NA
**GI—congenital diaphragmatic hernia (CDH) (HP:0000776)**	NA	NA	NA	NA	Yes, left sided	NA	Yes, left sided	NA	NA
**GU—hydronephrosis (HP:0000126)**	Yes, unspecified	NA	Yes, unspecified	No	NA	NA	NA	NA	NA
**GU—hydroureters (HP:0000072)**	NA	NA	NA	No	NA	NA	NA	NA	NA
**GU—recurrent urinary tract infections (HP:0000010)**	NA	NA	NA	NA	NA	NA	NA	NA	NA
**GU—vesicoureteral reflux (HP:0000076)**	NA	NA	Yes	No	NA	NA	NA	Yes	NA
**GU—structural anomalies (HP:0000119)**	Ectopic ureters	Ectopic ureter, side not specified	NA	No	Ectopic ureters; renal pelviectasis	Ectopic ureter	Right kidney dilatation	NA	Ectopic ureters; congenital urogenital sinus
**GU—functional complications (HP:0000025)**	NA	NA	NA	NA	NA	NA	NA	Chronic kidney disease, not specified	Chronic kidney disease, not specified
**Cardiovascular—structural anomalies (HP:0030680)**	Left ventricular noncompaction	Aortic coarctation; bicuspid aortic valve	Left ventricular noncompaction; ascending aortic dilatation; pulmonary artery stenosis	No	Aortic coarctation	Left ventricular noncompaction; aorta ascendens dilatation	Left ventricular noncompaction; aortic coarctation with postoperative restenosis; pulmonic vein stenosis with postoperative restenosis	Left ventricular noncompaction; atrial/ventricular septal defects; aortic coarctation; outpouching of the left cavernous internal carotid artery	Left ventricular noncompaction; aorta ascendens dilatation with aorta descendens narrowing; tricuspid regurgitation
**Cardiovascular—functional complications (HP:0011025)**	NA	Atrial and ventricular arrhythmia; ventricular fibrillation	NA	NA	NA	NA	Decreased left ventricular function	NA	Arterial hypertension
**Skeletal (HP:0000924)**	NA	Scoliosis	NA	NA	NA	NA	Scoliosis	Scoliosis	NA
**Clinical feature/field**	**CASE 10**	**CASE 11**	**CASE 12**	**CASE 13**	**CASE 14**	**CASE 15**	**CASE 16**	**CASE 17**	**CASE 18**
**Sex**	F	F	F	F	F	F	F	F	F
**Age at diagnosis**	14 years	9 months	NA	18 years	Prenatal	Prenatal	Postnatal	14 months	21 months
**Age at last evaluation**	14 years	9 months	21 months	20 years, deceased	12 months	TOP	<1 year	2 years + 8 months	6 years
**Development—intellectual (HP:0001249)**	Normal	Mild delay	Delayed	Normal	Normal	NA	NA	Normal	Normal
**Development—motor (HP:0001263)**	Normal	Mild delay	Delayed	NA	Normal	NA	NA	Normal	Normal
**Development—language (HP:0000750)**	Normal	Mild delay	Delayed	Slightly below average	Normal	NA	NA	Normal	Normal
**Cleft lip (HP:0410030)**	Yes, right sided	No	Yes	Yes, not specified	Yes, not specified	Bilateral	Unilateral, side not specified	Unilateral, left	No
**Cleft palate (HP:0000175)**	Yes, soft palate	Yes, not specified	Yes	Yes, not specified	Yes, not specified	Bilateral	Unilateral, side not specified	No	Unilateral, side not specified
**Hearing impairment (HP:0000365)**	NA	Bilateral, type not specified	NA	NA	Yes, mixed	NA	NA	NA	NA
**Pigmentary retinopathy (HP:0000580)**	NA	NA	NA	Yes	Yes	NA	NA	No	Yes
**GI—Intestinal malrotation (HP:0002566)**	No	Yes	NA	Yes	No	Yes	NA	Yes	NA
**GI—cholangitis (HP:0030151)**	NA	NA	NA	NA	NA	NA	NA	NA	NA
**GI—cholestasis (HP:0001396)**	NA	NA	NA	NA	NA	NA	NA	NA	NA
**GI—choledochal cyst (HP:0100890)**	NA	NA	NA	Yes	Yes	NA	NA	Yes	Yes
**GI—hepatomegaly (HP:0002240)**	NA	NA	NA	NA	NA	NA	NA	NA	NA
**GI—splenomegaly (HP:0001744)**	Yes	NA	NA	NA	NA	NA	NA	NA	NA
**GI—absent gallbladder (HP:0011467)**	No	No	NA	No	No	NA	NA	NA	Yes
**GI—liver fibrosis (HP:0001395)**	NA	NA	NA	NA	NA	NA	NA	Yes	NA
**GI—jaundice (HP:0000952)**	NA	NA	NA	NA	NA	NA	NA	NA	NA
**GI—Biliary atresia (HP:0005912)**	Yes	NA	NA	No	NA	NA	Yes	NA	NA
**GI—obstructive liver disease (HP:0005230)**	NA	NA	NA	NA	NA	NA	NA	NA	NA
**GI—liver biopsy/histology notes**	NA	NA	NA	Fatal intrahepatic cholangiocarcinoma	NA	NA	NA	NA	NA
**GI—congenital diaphragmatic hernia (CDH) (HP:0000776)**	NA	Yes, left sided	NA	NA	NA	Yes	NA	Yes	NA
**GU—hydronephrosis (HP:0000126)**	NA	NA	NA	Yes, not specified, secondary to uretral stenosis	Bilateral	NA	Bilateral	Unilateral, left	NA
**GU—hydroureters (HP:0000072)**	NA	NA	NA	NA	Bilateral	NA	Unilateral, left	NA	NA
**GU—recurrent urinary tract infections (HP:0000010)**	NA	NA	NA	NA	NA	NA	NA	NA	NA
**GU—vesicoureteral reflux (HP:0000076)**	NA	NA	NA	NA	Bilateral	NA	NA	NA	NA
**GU—structural anomalies (HP:0000119)**	Ectopic ureters; urogenital sinus; right renal artery stenosis with post-procedure renal artery restenosis	Abnormal renal morphology	NA	Uretral stenosis; partial bladder agenesis with urethral atresia; bicornuate noncommunicating uterus with hematosalpinx and hematometra; vaginal atresia	Small right renal cyst; ectopic ureters	Uterus, vagina and ovaries not identifiable	NA	NA	Cloacal anomaly
**GU—functional complications (HP:0000025)**	NA	NA	NA	Chronic kidney disease; kidney transplant	No	NA	NA	NA	NA
**Cardiovascular—structural anomalies (HP:0030680)**	Aortic dilatation	Atrial septal defect; patent ductus arteriosus	Congenital heart disease: Ebstein anomaly; thickened tricuspid valve with regurgitation	Patent ductus arteriosus; aortic coarctation	No	VSD; right sided aortic arch; double outlet right ventricle	Aortic coarctation	Aortic coarctation	Aortic coarctation
**Cardiovascular—functional complications (HP:0011025)**	Arterial hypertension	NA	Tricuspid regurgitation	No	Atrial ectopy	NA	No	No	No
**Skeletal (HP:0000924)**	NA	NA	Digit anomalies	Kyphosis (11 years); incomplete fusion of S1 posterior elements; Sprengel deformity; C2/C3 retrolisthesis	NA	NA	NA	NA	NA
**Clinical feature/field**	**CASE 19**	**CASE 20**	**CASE 21**	**CASE 22**	**CASE 23**	**CASE 24**	**CASE 25**	**CASE 26**	**CASE 27**
**Sex**	F	F	F	F	F	F	F	F	F
**Age at diagnosis**	12 months	Postnatal	4 years	19 months	Prenatal	Prenatal	Prenatal	Prenatal	9 months
**Age at last evaluation**	14 years	21 years, deceased	4 years	33 months	TOP	TOP	TOP	Fetal demise	9 months
**Development—intellectual (HP:0001249)**	Normal	Normal	Normal	Normal	NA	NA	NA	NA	Mild delay
**Development—motor (HP:0001263)**	Normal	Normal (initially delayed at 1 year)	Normal	Normal	NA	NA	NA	NA	Mild delay
**Development—language (HP:0000750)**	Normal	Normal	Normal	Normal	NA	NA	NA	NA	Mild delay
**Cleft lip (HP:0410030)**	Bilateral	Bilateral	Yes, not specified	No	Yes	Bilateral	Yes, right sided	Yes, not specified	No
**Cleft palate (HP:0000175)**	Bilateral	Bilateral	Yes, not specified	Yes, not specified	Yes	Bilateral	Yes, right sided	No	Yes, not specified
**Hearing impairment (HP:0000365)**	NA	Yes, conductive, bilateral	NA	Yes, sensorineural	NA	NA	NA	NA	Bilateral, type not specified
**Pigmentary retinopathy (HP:0000580)**	Yes	Yes	Yes	NA	NA	NA	NA	NA	NA
**GI—Intestinal malrotation (HP:0002566)**	Yes	Yes	NA	NA	NA	No	NA (no autopsy)	No	Yes
**GI—cholangitis (HP:0030151)**	No	No	NA	NA	NA	NA	NA	NA	NA
**GI—cholestasis (HP:0001396)**	Yes	Yes	Yes	NA	NA	NA	NA	NA	NA
**GI—choledochal cyst (HP:0100890)**	Yes, type 1	No	NA	NA	NA	NA	NA	NA	NA
**GI—hepatomegaly (HP:0002240)**	Yes	Yes	NA	NA	NA	NA	NA	NA	NA
**GI—splenomegaly (HP:0001744)**	Yes	Yes	NA	NA	NA	NA	NA	NA	NA
**GI—absent gallbladder (HP:0011467)**	NA	No	Yes	NA	NA	NA	NA	No	No
**GI—liver fibrosis (HP:0001395)**	Yes	Yes	NA	NA	NA	NA	NA	NA	NA
**GI—jaundice (HP:0000952)**	Yes	Yes	NA	NA	NA	NA	NA	NA	NA
**GI—Biliary atresia (HP:0005912)**	No	Yes	NA	NA	NA	NA	NA	NA	NA
**GI—obstructive liver disease (HP:0005230)**	NA	Yes	NA	NA	NA	NA	NA	NA	NA
**GI—liver biopsy/histology notes**	At 1 month: bile duct proliferation + fibrosis; at 7.5 months: cirrhosis with regenerating nodules + chronic portal inflammation with bile duct proliferation + lobular cholestasis	Fibrosis + porto-portal septa + marked ductular proliferation + polymorphonuclear infiltration	NA	NA	NA	NA	NA	NA	NA
**GI—congenital diaphragmatic hernia (CDH) (HP:0000776)**	NA	NA	NA	NA	Yes	Yes	Yes, left sided	Yes	Yes, left sided
**GU—hydronephrosis (HP:0000126)**	Bilateral	Bilateral	NA	NA	NA	Bilateral	NA	Yes, bilateral	NA
**GU—hydroureters (HP:0000072)**	Bilateral	Bilateral	NA	NA	NA	NA	NA	NA	NA
**GU—recurrent urinary tract infections (HP:0000010)**	Yes	Yes	NA	NA	NA	NA	NA	NA	NA
**GU—vesicoureteral reflux (HP:0000076)**	Yes, not specified	Bilateral	NA	NA	NA	NA	NA	NA	NA
**GU—structural anomalies (HP:0000119)**	Ectopic ureters; bladder exstrophy; vaginal atresia; urogenital sinus	Ectopic ureters; vaginal atresia	Ectopic ureters, bilateral	NA	NA	No	NA	Gingival atrophy	Abnormal renal morphology
**GU—functional complications (HP:0000025)**	Chronic renal insufficiency (KDIGO 2)	Chronic renal insufficiency (KDIGO 2)	NA	NA	NA	NA	NA	NA	NA
**Cardiovascular—structural anomalies (HP:0030680)**	Patent ductus arteriosus; aortic coarctation	Patent ductus arteriosus; patent foramen ovale; coarctatio aortae; partial anomalous pulmonary venous return; dilated ascending aorta (27 mm); bicuspid aortic valve; tricuspid valve regurgitation (3/4); mitral valve regurgitation (2/4)	Patent ductus arteriosus	Mild aortic dilatation	Normal	Atrioventricular septal defect; patent ductus arteriosus	NA	NA	Atrial septal defect; patent ductus arteriosus
**Cardiovascular—functional complications (HP:0011025)**	No	Systolic murmur	NA	NA	Normal	NA	NA	NA	NA
**Skeletal (HP:0000924)**	Osteoporosis	No	NA	NA	dolichocephaly and turricephaly	NA	NA	NA	NA
**Clinical feature/field**	**CASE 28**	**CASE 29**	**CASE 30**	**CASE 31**	**CASE 32**	**CASE 33**	**CASE 34**	**CASE 35** (current case)	
**Sex**	F	F	F	F	F	F	F	F	
**Age at diagnosis**	34 years	19 months	2 years	Prenatal	NA	Prenatal	NA	3 years	
**Age at last evaluation**	36 years	3 years	7 years	TOP	4 years	TOP	7 years	3 years, deceased	
**Development—intellectual (HP:0001249)**	Normal	Normal	Normal	NA	Delayed at 3 years	NA	Delayed	Normal	
**Development—motor (HP:0001263)**	Normal	Normal	Normal	NA	Delayed at 3 years	NA	Delayed	Normal	
**Development—language (HP:0000750)**	Normal	Normal	Normal	NA	Delayed at 3 years	NA	Delayed	Normal	
**Cleft lip (HP:0410030)**	No	No	No	Bilateral	Unilateral, left	Yes, left sided	No	No	
**Cleft palate (HP:0000175)**	No	No	No	Bilateral	No	Yes, left sided	No	No	
**Hearing impairment (HP:0000365)**	NA	NA	Yes, sensorineural, right sided	NA	Yes, unspecified	NA	NA	No	
**Pigmentary retinopathy (HP:0000580)**	NA	NA	NA	NA	NA	NA	NA	No	
**GI—Intestinal malrotation (HP:0002566)**	No	No	Yes	NA	Yes	NA	NA	No	
**GI—cholangitis (HP:0030151)**	No	No	No	NA	NA	NA	NA	No	
**GI—cholestasis (HP:0001396)**	No	No	Yes	NA	NA	NA	NA	Yes	
**GI—choledochal cyst (HP:0100890)**	Yes	Yes	No	Yes	NA	NA	NA	No	
**GI—hepatomegaly (HP:0002240)**	No	No	No	NA	NA	NA	NA	Yes	
**GI—splenomegaly (HP:0001744)**	No	No	No	NA	NA	NA	NA	Yes	
**GI—absent gallbladder (HP:0011467)**	No	No	No	No	NA	NA	NA	Yes	
**GI—liver fibrosis (HP:0001395)**	No	No	No	NA	NA	NA	NA	Yes	
**GI—jaundice (HP:0000952)**	Yes	Yes	No	NA	NA	NA	NA	Yes	
**GI—Biliary atresia (HP:0005912)**	Yes	No	No	NA	NA	NA	Yes	No	
**GI—obstructive liver disease (HP:0005230)**	No	No	No	NA	NA	NA	NA	No	
**GI—liver biopsy/histology notes**	NA	NA	Bilirubinostasis	NA	NA	NA	NA	Cholestasis and paucity of interlobular bile ducts, Portal fibrosis with a few septa and no evidence of cirrhosis, Mild intrahepatocytic and Kupffer cell hemosiderosis.	
**GI—congenital diaphragmatic hernia (CDH) (HP:0000776)**	NA	NA	NA	NA	NA	Yes	NA	No	
**GU—hydronephrosis (HP:0000126)**	Bilateral	Bilateral	Bilateral	Bilateral	Bilateral	Yes, left sided	NA	No	
**GU—hydroureters (HP:0000072)**	Bilateral	Bilateral	Bilateral	Bilateral	NA	Yes, left sided	NA	No	
**GU—recurrent urinary tract infections (HP:0000010)**	Yes	Yes (E. cloacae)	Yes	NA	NA	NA	NA	No	
**GU—vesicoureteral reflux (HP:0000076)**	Yes	Yes	Yes	NA	NA	NA	NA	No	
**GU—structural anomalies (HP:0000119)**	Ectopic ureters; bicornuate uterus; vaginal atresia	Ectopic ureters; uterus didelphys; distal vaginal atresia	Ectopic ureters; hypoplastic uterus; vaginal atresia	NA	Ectopic ureters	NA	Bicornuate uterus; urogenital sinus anomaly	No	
**GU—functional complications (HP:0000025)**	Chronic kidney disease (KDIGO 3a)	No	No	NA	NA	NA	NA	No	
**Cardiovascular—structural anomalies (HP:0030680)**	NA	No	Patent foramen ovale; biventricular hypertrophy; aortic coarctation; dilated ascending aorta (13 mm); bicuspid aortic valve; tricuspid valve regurgitation (1/4)	Dextrocardia	Patent foramen ovale	Large VSD	Abnormal heart morphology	No	
**Cardiovascular—functional complications (HP:0011025)**	NA	No	Supraventricular tachycardia	NA	NA	NA	NA	No	
**Skeletal (HP:0000924)**	No	No	Sacral dimple	NA	NA	NA	NA	sacrococcygeal agenesis	
**Other**							Abnormal pulmonary interstitial morphology, bronchomalacia, restrictive ventilatory defect, abducens palsy and global developmental delay.	Possible immunodeficiency, suggested by recurrent respiratory infections and sepsis.	

F: female, GI: gastrointestinal, GU: genitourinary, NA: not applicable, TOP: termination of pregnancy.

**Table 2:** Molecular spectrum of *MED12* variants in all reported patients with Hardikar syndrome in the literature, including the present case.

Cases	HGVS NC_000023.11:	cDNA NM_005120.3:	Protein	ACMG Classification	ACMG Scoring	Variant Type	Inheritance	Sex	References
**CASE 1**	g.71121146del	c.729delG	p.(Met243Ilefs*13)	Pathogenic	PVS1, PS2, PM2	Frameshift	De Novo	F	Strong et al. [8]
**CASE 2**	g.71123713del	c.1737del	p.(Met580Cysfs*2)	Pathogenic	PVS1, PS2, PM2	Frameshift	De Novo	F	
**CASE 3**	g.71124338_71124405dup	c.1924_1974+17dup	p.?	Likely Pathogenic	PS2, PM2	Splice	De Novo	F	
**CASE 4**	g.71126059C>T	c.2446C>T	p.(Arg816*)	Pathogenic	PVS1, PS2, PM2	Nonsense	De Novo	F	
**CASE 5**	g.71129860G>A	c.3867+5G>A	p.?	Likely Pathogenic	PS2, PM2	Splice	De Novo	F	
**CASE 6**	g.71131549G>A	c.4048-1G>A	p.?	Pathogenic	PVS1, PM2, PM6	Splice	NA	F	
**CASE 7**	g.71132072G>A	c.4120-1G>A	p.?	Pathogenic	PVS1, PS2, PM2	Splice	De Novo	F	
**CASE 8**	g.71136366G>A	c.5111G>A	p.(Trp1704*)	Pathogenic	PVS1, PS2, PM2	Nonsense	De Novo	F	
**CASE 9**	g.71136390_ 71136393dup	c.5135_5138dup	p.(Val1714Alafs*37)	Pathogenic	PVS1, PS2, PM2	Frameshift	De Novo	F	
**CASE 10**	g.71136401C>T	c.5146C>T	p.(Arg1716*)	Pathogenic	PVS1, PS2, PM2	Nonsense	De Novo, Mosaic	F	
**CASE 11**	g.71140759C>T	c.6169C>T	p.(Gln2057*)	Pathogenic	PVS1, PS2, PM2	Nonsense	De Novo	F	
**CASE 12**	g.71137942dup	c.6043dup	p.(Arg2015Lysfs*36)	Pathogenic	PVS1, PM2, PM6	Frameshift	NA	F	Wang et al. [12]
**CASE 13**	g.71130185C>T	c.4018C>T	p.(Gln1340*)	Pathogenic	PVS1, PS2	Nonsense	De Novo, Mosaic	F	Pillai et al. [13]
**CASE 14**	g.71125144C>T	c.2224C>T	p.(Gln742*)	Pathogenic	PVS1, PS2, PM2	Nonsense	De Novo	F	
**CASE 15**	g.71140786C>T	c.6196C>T	p.(Gln2066*)	Pathogenic	PVS1, PS2, PM2	Nonsense	De Novo	F	Faergerman et al. [14]
**CASE 16**	g.71119803C>T	c.322C>T	p.(Arg108*)	Pathogenic	PVS1, PS2, PM2	Nonsense	De Novo, Mosaic	F	Li et al. [1]
**CASE 17**	g.71125127_ 71125130del	c.2207_2210del	p.(Thr736Ilefs*43)	Pathogenic	PVS1, PS2, PM2	Frameshift	De Novo	F	
**CASE 18**	g.71126462dup	c.2663dup	p.(Leu889Profs*11)	Pathogenic	PVS1, PS2, PM2	Frameshift	De Novo	F	
**CASE 19**	g.71135131_ 71135134delinsCCAGCA	c.4903_4906delinsCCAGCA	p.(Val1635Profs*61)	Pathogenic	PVS1, PM2, PM6	Frameshift	De Novo	F	
**CASE 20**	g.71136366G>A	c.5111G>A	p.(Trp1704*)	Pathogenic	PVS1, PS2, PM2	Nonsense	De Novo	F	
**CASE 21**	g.71137257C>A	c.5622C>A	p.(Tyr1874*)	Pathogenic	PVS1, PS2, PM2	Nonsense	De Novo	F	
**CASE 22**	g.71140759C>T	c.6169C>T	p.(Gln2057*)	Pathogenic	PVS1, PS2, PM2	Nonsense	De Novo	F	
**CASE 23**	g.71118855T>G	c.99+2T>G	p.?	Likely Pathogenic	PVS 1 PM2 PS2	Splice	De Novo	F	Ramachandran et al. [11]
**CASE 24**	g.71136978_TACAGGCAdel	c.5500_5507del	p. (Tyr1834Argfs*58)	Pathogenic	PVS1 PS2 PM2	Frameshift	De Novo	F	Dai et al. [15]
**CASE 25**	arr[GRCh38] Xq13.1(71,043,894–71,344,313)x1	-	-	Likely Pathogenic	-	Deletion (large CNV)	De Novo	F	Wu et al. [16]
**CASE 26**	g.71137260del	c.5625del	p.(Gly1876Glufs*7)	Pathogenic	PVS1, PM2, PP5	Frameshift	De Novo	F	Kao et al. [10]
**CASE 27**	g.71140759C>T	c.6169C>T	p.(Gln2057*)	Pathogenic	PVS1, PM2, PP5	Nonsense	De Novo	F	
**CASE 28**	g.71121435C>T	c.844C>T	p.(Arg282*)	Likely pathogenic	PVS1, PM2	Nonsense	De Novo, Mosaic	F	Warmoeskerken et al. [17]
**CASE 29**	g.71121435C>T	c.844C>T	p.(Arg282*)	Likely pathogenic	PVS1, PM2	Nonsense	Maternal	F	Warmoeskerken et al. [17]
**CASE 30**	g.71130042del	c.3875del	p.(Val1292Glufs*21)	Likely pathogenic	PVS1, PM2	Frameshift	De Novo	F	Warmoeskerken et al. [17]
**CASE 31**	g.71127962G>A	c.3051G>A	p.(Trp1017*)	Likely pathogenic	PVS1, PM2	Nonsense	De Novo	F	Warmoeskerken et al. [17]
**CASE 32**	g.71129189del	c.3551delA	p.(Gln1184fs)	Likely pathogenic	PVS1, PM2	Frameshift	De Novo	F	Munabi et al. [18]
**CASE 33**	g.71132844G>C	c.4416-1G>C	p. ?	Likely pathogenic	PVS1, PM2, PP5	Splice	De Novo	F	Taniguchi et al. [19]
**CASE 34**	g.71129456_71131855del	-	p.(Gly1967Valfs*12)	Likely pathogenic	-	Frameshift	De Novo	F	Decipher
**CASE 35**	g.71130030C>G	c.3868-5C>G	p. ?	VUS	PP3, PM2, PS2	Splice	De Novo	F	El Kahy et al. (*Current Case*)

ACMG: American College of Medical Genetics and Genomics [20], CNV: Copy Number Variant; F: female, HGVS: Human Genome Variation Society [21] (https://hgvs-nomenclature.org/, accessed on 12 May 2026), NA: Not Applicable., VUS: Variant of Uncertain Significance.

## Data Availability

The data that supports the findings of this study are available from the corresponding author upon reasonable request.
